# Real‐world study of lazertinib as second‐line or greater treatment in advanced non‐small cell lung cancer

**DOI:** 10.1111/1759-7714.15337

**Published:** 2024-05-27

**Authors:** Jeong Uk Lim, Kyuhwan Kim, Kyu Yean Kim, Hye Seon Kang, Ah. Young Shin, Chang Dong Yeo, Sung Kyoung Kim, Chan Kwon Park, Sang Haak Lee, Seung Joon Kim

**Affiliations:** ^1^ Division of Pulmonary, Allergy, and Critical Care Medicine, Department of Internal Medicine, Yeouido St. Mary's Hospital, College of Medicine The Catholic University of Korea Seoul Republic of Korea; ^2^ Division of Pulmonary, Allergy, and Critical Care Medicine, Department of Internal Medicine, Seoul St. Mary's Hospital, College of Medicine The Catholic University of Korea Seoul Republic of Korea; ^3^ Division of Pulmonary, Critical Care Medicine, Department of Internal Medicine, Uijeongbu St. Mary's Hospital, College of Medicine The Catholic University of Korea Uijeongbu‐si Republic of Korea; ^4^ Division of Pulmonary and Critical Care Medicine, Department of Internal Medicine, Bucheon St. Mary's Hospital, College of Medicine The Catholic University of Korea Bucheon‐si Republic of Korea; ^5^ Division of Pulmonary and Critical Care Medicine, Department of Internal Medicine, Incheon St. Mary's Hospital, College of Medicine The Catholic University of Korea Incheon Republic of Korea; ^6^ Division of Pulmonary, Critical Care and Sleep Medicine, Department of Internal Medicine, Eunpyeong St. Mary's Hospital, College of Medicine The Catholic University of Korea Seoul Republic of Korea; ^7^ Division of Pulmonary and Critical Care Medicine, Department of Internal Medicine, St. Vincent's Hospital, College of Medicine The Catholic University of Korea Suwon‐si Republic of Korea; ^8^ Postech‐Catholic Biomedical Engineering Institute, Songeui Multiplex Hall, College of Medicine The Catholic University of Korea Seoul Republic of Korea

**Keywords:** *EGFR* mutation, lazertinib, non‐small cell lung cancer, targeted therapy

## Abstract

**Background:**

Lazertinib is an oral, third‐generation EGFR‐TKI, which specifically targets the *EGFR* T790M mutation along with activating mutations Ex19del and L858R. More real‐world data are needed to evaluate its efficacy and safety in treating locally advanced and metastatic non‐small cell lung cancer (NSCLC) following prior EGFR TKI treatment.

**Methods:**

This multicenter retrospective study was conducted at seven university hospitals affiliated to the Catholic Medical Center (CMC) in Korea. A clinical data warehouse (CDW) platform was used to access and extract information.

**Results:**

A total of 48 patients were assessed. The majority were female (75%) and diagnosed with adenocarcinoma (95.8%). All patients had the *EGFR* mutation at diagnosis, 27 (56.3%) had the exon 19 deletion, 20 (41.7%) had the L858R mutation, and one (2.0%) had the exon 18 mutation. The median progression‐free survival (PFS) was 15.4 months. At 6, 12, and 18 months, PFS rates were 79.1%, 53.6%, and 27.3%, respectively. When PFS was analyzed by prior TKI duration (<18 months vs. >18 months), significant differences were noted at the 6 and 9‐month mark (*p* = 0.013 and *p* = 0.010, respectively). In multivariate analysis for PFS, only prior TKI duration and ECOG score showed statistical significance (*p* = 0.026 and *p* = 0.049, respectively). In the multivariate analysis for OS, ECOG score showed statistical significance (*p* = 0.006). Among 48 patients, 34 (70.8%) experienced adverse events (AEs) related to lazertinib. The most frequent AEs were skin reaction (29.8%), diarrhea (21.3%), and peripheral neuropathy (20.8%).

**Conclusions:**

The results suggest that lazertinib is effective in second or more line settings, with tolerable safety profile. More patient data are necessary to find possible prognostic markers associated with patient outcome.

## Graphical abstract

Lazertinib is an oral, third‐generation EGFR‐TKI, and it specifically targets the *EGFR* T790M mutation along with activating mutations Ex19del and L858R. This multicenter retrospective study was conducted at seven university hospitals. When PFS was analyzed by prior TKI duration (<18 months vs. >18 months), significant differences were noted at the 6 and 9‐month marks (*p* = 0.013 and *p* = 0.010, respectively).

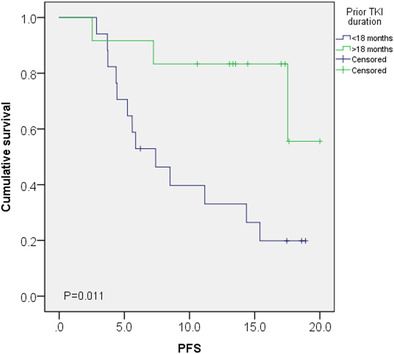

## INTRODUCTION

Epidermal growth factor receptor (EGFR)‐tyrosine kinase inhibitors (TKIs) are the standard treatment for non‐small cell lung cancer (NSCLC) with *EGFR* activating mutations (e.g., Ex19del or L858R).^1^, ^2^ However, many patients develop resistance to first‐generation (gefitinib, erlotinib) or second‐generation (afatinib, dacomitinib) EGFR‐TKIs. The primary cause of acquired resistance is the *EGFR* exon 20 T790M point mutation.^3^, ^4^ Thus, there is a need to develop third‐generation TKIs that specifically target the *EGFR* T790M mutation.

Lazertinib (LECLAZA) is an oral, third‐generation EGFR‐TKI being developed by Yuhan and Janssen Biotech for treating NSCLC. It is a brain‐penetrant, irreversible EGFR‐TKI that targets the *EGFR* T790M mutation, activates mutations Ex19del and L858R, and spares wild‐type *EGFR*.

An open‐label, multicenter, phase 1–2 study in Korea investigated the use of lazertinib in patients with advanced NSCLC harboring an activating *EGFR* mutation and progressing after EGFR TKI treatment. Lazertinib demonstrated promising efficacy, with an objective response rate (ORR) of 54%. Adverse events were generally manageable, and no dose‐limiting toxicities were observed.^5^ Lazertinib received its first approval for treating patients with *EGFR* T790M mutation‐positive locally advanced or metastatic NSCLC who previously received EGFR‐TKI therapy.^6^ The LASER 301 study shows the possibility of lazertinib as a first‐line EGFR TKI in metastatic NSCLC.^7^

However, there are few studies available.^8^, ^9^ The aim of this study was to evaluate the efficacy and safety profile of lazertinib in patients with locally advanced and metastatic NSCLC after prior EGFR TKI treatment.

## METHODS

### Patient selection

**FIGURE 1:**
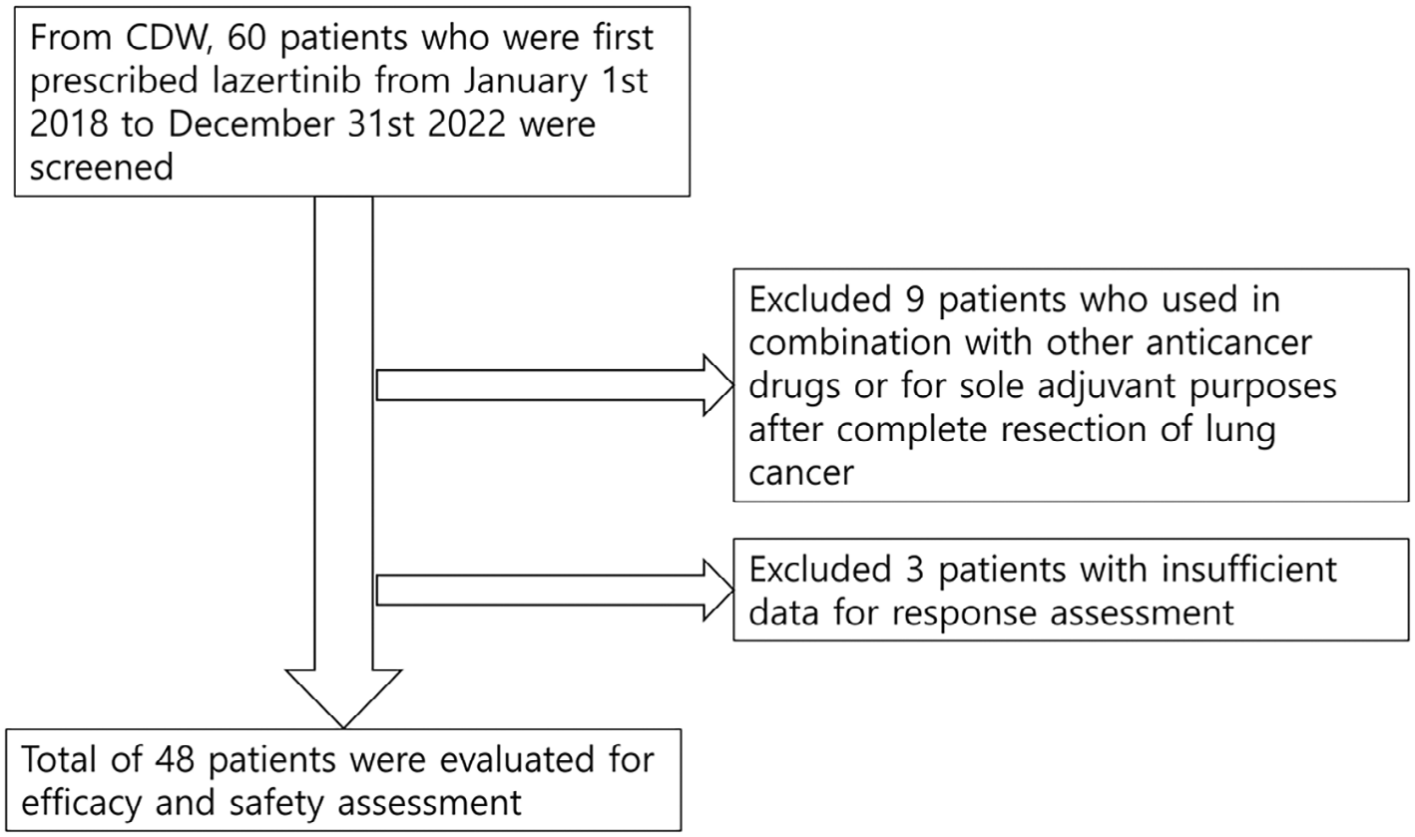
Flow diagram of study patient enrollment.

This multicenter retrospective study was conducted at seven Catholic Medical Center (CMC) hospitals in Korea. Data from cases of patients who used lazertinib were evaluated. A clinical data warehouse (CDW) platform was used to access and extract information. The CDW we used, which incorporates seven affiliated CMC hospitals in Korea, is a data platform that collects and distributes clinical data to researchers.^10^, ^11^, ^12^ Our study utilized a database containing more than 15 million completely anonymized electronic medical records. The target patient population consisted of adults aged 18 years or older with International Classification of Diseases, 10th revision (ICD‐10) codes indicating lung cancer (ICD codes starting with C34). The inclusion criteria were patients who were prescribed lazertinib for at least one month, while exclusion criteria were use of lazertinib in combination with other anticancer drugs or for adjuvant purposes after complete resection of lung cancer (Figure 1). The study protocol received approval from the Institutional Review Board of The Catholic University of Korea (no. SC23WISI0079). As anonymized clinical data were utilized, the need for informed consent was waived.

### Efficacy assessments

Our primary goal was to determine the median progression‐free survival (PFS), defined as the time from starting lazertinib to disease exacerbation or death while on lazertinib. Our secondary goals were to assess the median overall survival (OS, the time from starting lazertinib to death from any cause) and ORR. Intracranial ORR and PFS were not examined because few patients experienced intracranial metastases. Tumor response was assessed every 8–12 weeks with standard scans, including chest computed tomography (CT) and brain magnetic resonance imaging (MRI). The response was judged based on clinical evaluation and the RECIST version 1.1 criteria.^13^

### Statistical analysis

We employed descriptive statistics to show baseline patient data. We assessed relationships among categorical items using Chi‐square or Fisher's tests. We analyzed the correlations between continuous and categorical data points using *t*‐tests. To assess patient survival durations, we used Kaplan–Meier plots. We assessed differences in the timing of events through log‐rank tests. When median PFS or OS was analyzed using Kaplan Meier plots, only the patients who were censored during the observation period or who survived without any events for more than 12 months were included, ensuring a sufficient observation period. For evaluation of hazard ratios, we performed both univariate and multivariate analyses of the Cox proportional hazards. For all statistical evaluations, *p*‐values under 0.05 were considered statistically significant. All computations were executed using SPSS version 23.

## RESULTS

### Patient characteristics

We evaluated 48 patients with a median age of 69.1 years, 75% of whom were female. Regarding performance, 52.1%, 37.5%, and 10.4% of the patients showed ECOG score of 0, 1, and ≥2, respectively. Within our total patient group, 46 (95.8%) were diagnosed with adenocarcinoma. The remaining two patients (4.2%) were diagnosed with squamous cell type carcinoma. All patients had the *EGFR* mutation at diagnosis, 27 (56.3%) had the exon 19 deletion, 20 (41.7%) had the L858R mutation, and one (2.0%) had the exon 18 mutation (Table 1).

**TABLE 1:** Clinical characteristics of the enrolled patients.

Parameters	Number (*n* = 48)
Age, years (median)	69.1 (40–90)
Sex	
Male	12 (25.0)
Female	36 (75.0)
ECOG performance status	
0	25 (52.1)
1	18 (37.5)
2 or more	5 (10.4)
Tumor histology	
Adenocarcinoma	46 (95.8)
Squamous	2 (4.2)
*EGFR* mutation status	
Exon 19 deletion	27 (56.3)
L858R	20 (41.7)
Exon 18	1 (2.0)
EGFR T790M status	
Positive	38 (92.6)
Negative	3 (7.3)
Blood EGFR test	
T790M positive	21 (63.6)
T790M negative	12 (36.4)
Brain metastasis	
Yes	9 (18.7)
No	39 (81.3)
AJCC stage	
III	3 (6.2)
IV	45 (93.8)
Previous lines of systemic therapy	
1	42 (87.5)
2 or more	6 (12.5)
Patients who underwent complete resection	10 (20.8)
Treatment immediately preceding lazertinib	
Gefitinib	18 (37.5)
Erlotinib	8 (16.7)
Afatinib	17 (35.4)
Dacomitinib	3 (6.3)
Platinum chemotherapy	1 (2.1)
Immunotherapy	1 (2.1)
EGFR TKI treatment duration, median, range (months)	16.8 (0.9–57.4)
Gefitinib (18)	15.4 (0.9–47.6)
Erlotinib (8)	17.5 (2.3–57.4)
Afatinib (18)	18.3 (8.4–52.3)
Dacomitinib (3)	5.3 (1.3–15.3)
Brain metastasis at diagnosis	9 (18.8)
Bone metastasis at diagnosis	22 (45.8)
Intrathoracic metastasis, M1a	33 (68.8)

Abbreviations: AJCC, American Joint Committee on Cancer; ECOG, Eastern Cooperative Oncology Group; EGFR‐TKI, epidermal growth factor receptor‐tyrosine kinase inhibitor.

### Outcomes

**FIGURE 2:**
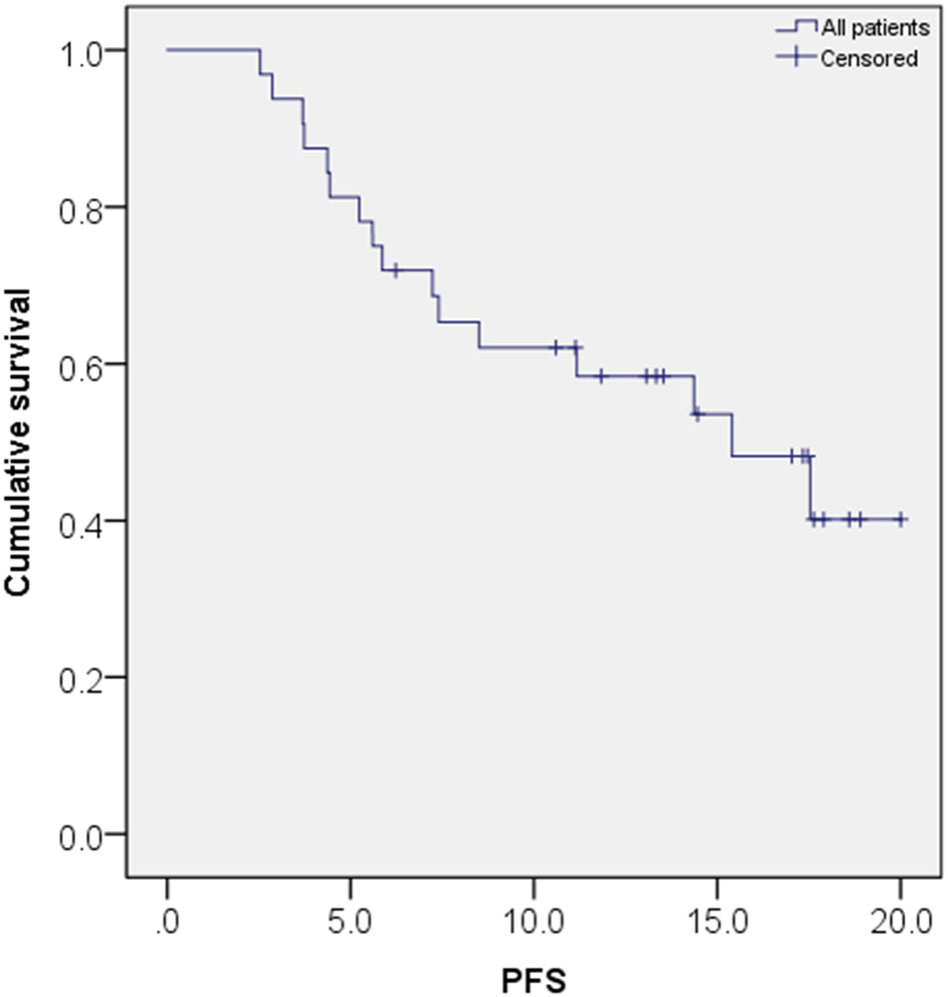
Progression‐free survival of all enrolled patients.

The median PFS of the patients was 15.4 months (95% CI: 8.7–22.1 months). At the six‐month follow up, the PFS rate was 79.1% (9 of 43 patients), at 12 months it was 53.6% (15 of 28 patients), and by 18 months, it had decreased to 27.3% (6 of 22 patients). The median OS was not reached (Table 2) (Figure 2).

**TABLE 2:** Clinical outcomes of the enrolled patients.

Treatment parameter	Values
Objective response rate, no. (%)	47.9%
Complete response	0 (0)
Partial response	23 (47.9)
Stable disease	16 (33.3)
Progressive disease	5 (10.4)
Not evaluable	4 (8.4)
Disease control rate (%)	81.3%
Median PFS, months (95% CI)	15.4 (8.7–22.1)
Lazertinib overall survival	Not reached
PFS rate %	
At 6 months	34/43 (79.1)
At 12 months	15/28 (53.6)
At 18 months	6/22 (27.3)

Abbreviations: CI, confidence interval; PFS, progression‐free survival.

**FIGURE 3:**
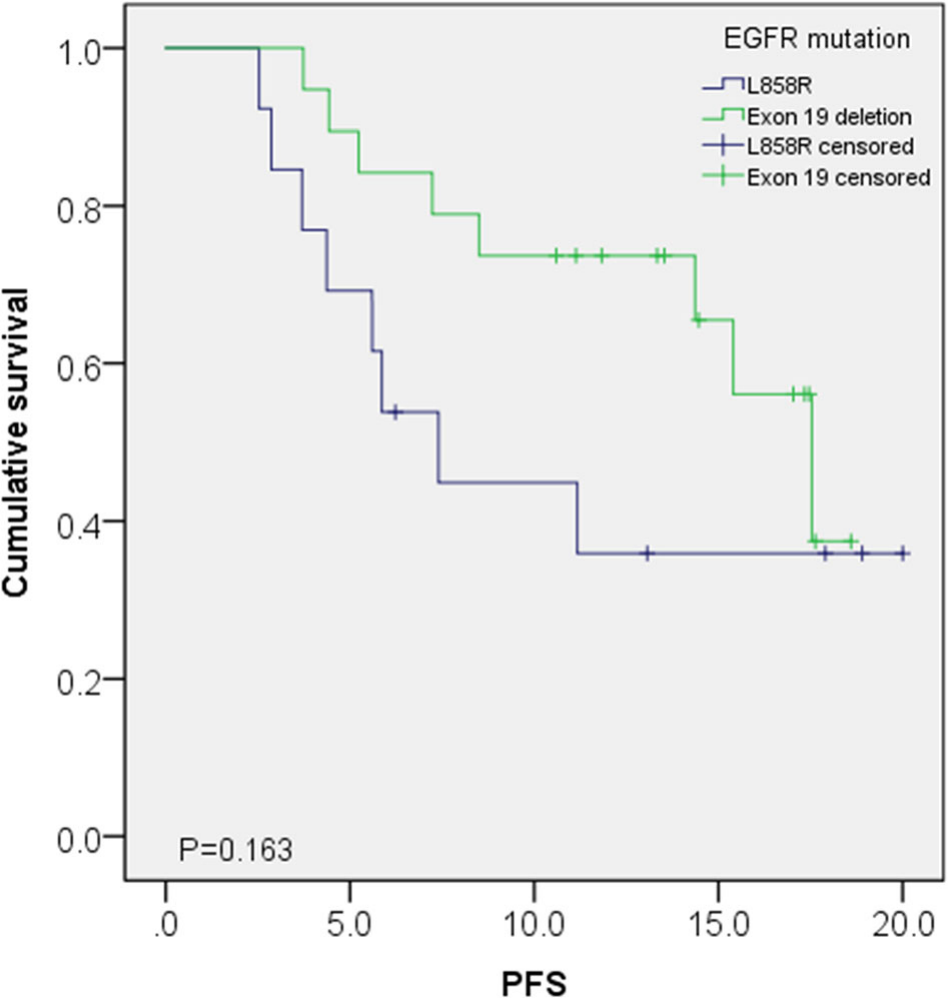
Comparison of progression‐free survival between L858R mutation and exon 19 deletion.

We performed PFS comparison based on *EGFR* mutation type. The median PFS did not show a significant difference between patients with the L858R mutation and those with the exon 19 deletion, though the exon 19 deletion subgroup tended to have superior PFS at 6 and 9 months (*p* = 0.090 and *p* = 0.063, respectively) (Figure 3). However, by the 12‐month follow up, we did not observe a difference in PFS between the groups (Table 3).

**TABLE 3:** Comparison of PFS between L858R mutation and exon 19 deletion.

Clinical outcome	L858R mutation	Exon 19 deletion	*p*‐value
Median PFS (month, 95% CI)	5.9 (1.8–10.0)	15.4 (11.5–19.3)	0.235
6 months PFS (*n* = 43)	12/18 (66.7%)	22/25 (88.0%)	0.090
12 months PFS (*n* = 28)	4/12 (33.3%)	11/16 (68.8%)	0.063
18 months PFS (*n* = 22)	3/11 (27.3%)	3/11 (27.3%)	1.000

Abbreviation: CI, confidence interval; PFS, progression‐free survival.

**FIGURE 4:**
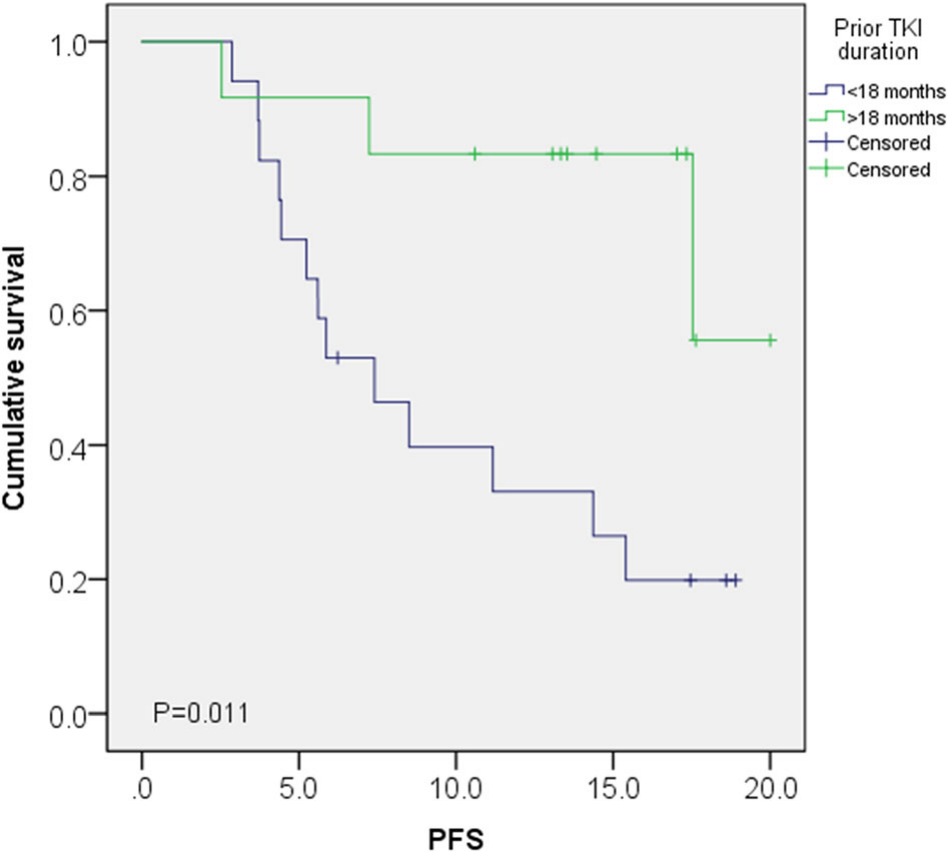
Comparison of progression‐free survival between patients under prior tyrosine kinase inhibitor (TKI) <18 months versus TKI >18 months.

Comparisons of PFS based on ECOG score at lazertinib initiation showed significant differences at the six‐ and 12‐month follow up (*p* = 0.005 and *p* = 0.049, respectively). The median PFS also showed a significant difference (*p* = 0.011) (Table 4). When analyzed in terms of prior TKI duration (<18 months vs. >18 months), significant differences were noted at six and 9 months (*p* = 0.013 and *p* = 0.010, respectively). While there was a significant difference in median PFS, it was not reached in the group with a TKI duration exceeding 18 months (*p* = 0.011) (Table 5) (Figure 4). Comparing groups stratified by T790M positivity on liquid biopsy, no significant differences were observed in median PFS or in PFS rates at 6, 12, and 18 months.

**TABLE 4:** Comparison of PFS between ECOG 0–1 versus 2–3.

Clinical outcome	ECOG 0–1	ECOG 2–3	*p*‐value
Median PFS (month, 95% CI)	17.5 (12.5–22.6)	4.4 (3.7–5.1)	0.011
6 months PFS (*n* = 43)	33/39 (84.6%)	1/4 (25.0%)	0.005
12 months PFS (*n* = 28)	15/25 (60.0%)	0/3 (0%)	0.049
18 months PFS (*n* = 22)	6/19 (31.6%)	0/3 (0%)	0.254

Abbreviations: ECOG, Eastern Cooperative Oncology Group; PFS, progression‐free survival.

**TABLE 5:** Comparison of PFS between patients under prior TKI <18 months versus TKI >18 months.

Clinical outcome	Prior TKI duration <18 months	Prior TKI duration >18 months	*p*‐value
Median PFS (month, 95% CI)	7.4 (3.7–11.0)	NR	0.011
6 months PFS (*n* = 42)	14/22 (63.6%)	19/20 (95.0%)	0.013
12 months PFS (*n* = 27)	5/16 (31.3%)	9/11 (81.8%)	0.010
18 months PFS (*n* = 21)	3/16 (18.8%)	2/5 (40.0%)	0.330

Abbreviations: PFS, progression‐free survival; TKI, tyrosine kinase inhibitor.

We performed Cox regression analyses for PFS and OS. In our univariate analysis for PFS, only prior TKI duration and ECOG score at baseline were significant factors (*p* = 0.011 and *p* = 0.006, respectively). When we entered these two factors into the multivariate analysis, they retained their statistical significance (*p* = 0.026 and *p* = 0.049, respectively) (Table 6). In our univariate analysis for OS, only the ECOG score showed statistical significance (*p* = 0.001). When we entered the ECOG score along with the prior TKI duration in the multivariate analysis, only the ECOG score retained statistical significance (*p* = 0.006) (Table 7).

**TABLE 6:** Cox regression for progression‐free survival (observation time at least 6 months) (*n* = 43).

		Univariate			Multivariate		
Parameter		HR	CI	*p*‐value	HR	CI	*p*‐value
Gender	Male (11)	1					
	Female (32)	1.005	0.320–3.160	0.993			
Age at diagnosis	Total *n* = 43	1.002	0.958–1.048	0.944			
Mutation	L858R mutation (18)	1					
	Exon 19 (25)	0.567	0.211–1.522	0.260			
Prior lines	One (38)	1					
	More than one (5)	0.934	0.210–4.152	0.934			
Prior TKI used	Gefitinib (16)	1					
	Erlotinib (8)	0.984	0.246–3.939	0.981			
	Afatinib (17)	0.989	0.332–2.950	0.984			
	Dacomitinib (2)	0.988	N/A	0.988			
Prior TKI duration	<18 months (22)	1		0.011	1		0.026
	>18 months (20)	0.196	0.056–0.690		0.232	0.064–0.843	
s/p complete resection	No history of complete resection (35)	1					
	Recur after complete resection (8)	0.574	0.130–2.538	0.464			
Blood T790M mutation at lazertinib initiation	None (12)	1					
	Positive (21)	0.908	0.265–3.107	0.877			
AJCC stage	Stage III (3)	1					
	Stage IV (40)	1.604	0.210–12.233	0.648			
Brain metastasis	None (34)	1					
	Yes (9)	1.084	0.307–3.831	0.900			
Intrathoracic metastasis (M1a)	None (14)	1					
	Yes (29)	2.182	0.618–7.702	0.225			
Smoking history	Never (32)	1					
	Ever (10)	0.5527	0.155–2.003	0.370			
ECOG	0–1	1			1		0.049
	2 or more	7.554	1.803–31.649	0.006	4.304	1.003–18.466	

Abbreviations: AJCC, American Joint Committee on Cancer; CI, confidence interval; ECOG, Eastern Cooperative Oncology Group; HR, hazard ratio; TKI, tyrosine kinase inhibitor.

**TABLE 7:** Cox regression for lazertinib overall survival (observation time at least 6 months).

		Univariate			Multivariate		
Parameter		HR	CI	*p*‐value	HR	CI	*p*‐value
Gender	Male (11)	1		0.369			
	Female (32)	N/A	N/A				
Age at diagnosis	Total *n* = 43	1.025	0.957–1.098	0.475			
Mutation	L858R mutation (18)	1		0.257			
	Exon 19 (25)	0.419	0.093–1.886				
Prior lines	One (38)	1		0.482			
	More than one (5)	0.038	N/A				
Prior TKI used	Gefitinib (16)	1		0.227			
	Erlotinib (8)	0.537	0.060–4.812	0.578			
	Afatinib (17)	0.204	0.023–1.839	0.157			
	Dacomitinib (2)	3.979	0.387–40.894	0.245			
Prior TKI duration	<18 months (22)	1		0.095	1		0.306
	>18 months (20)	0.164	0.020–1.366		0.306	0.032–2.949	
Prior complete resection	No history of complete resection (35)	1		0.867			
	Recur after complete resection (8)	0.835	0.100–6.955				
Blood T790M mutation at lazertinib initiation	None (12)	1		0.460			
	Positive (21)	2.287	0.255–20.497				
AJCC stage	Stage III (3)	1					
	Stage IV (40)	24.785	N/A				
Brain metastasis	None (34)	1		0.444			
	Yes (9)	1.909	0.364–10.008				
Intrathoracic metastasis (M1a)	None (14)	1		0.351			
	Yes (29)	2.747	0.328–22.998				
Smoking history	Never (32)	1		0.314			
	Ever (10)	0.028	N/A				
ECOG	0–1	1		0.001	1		
	2 and 3	45.619	4.560–456.407		28.047	2.610–301.376	0.006

Abbreviations: AJCC, American Joint Committee on Cancer; CI, confidence interval; ECOG, Eastern Cooperative Oncology Group; HR, hazard ratio; TKI, tyrosine kinase inhibitor.

### Safety

Among 48 patients, 34 (70.8%) experienced adverse events (AEs) related to lazertinib. The most frequent AEs were skin reaction (29.8%), diarrhea (21.3%), and peripheral neuropathy (20.8%). Other less frequent AEs reported were tachycardia, chest pain, dizziness, vomiting, thrombocytopenia, cardiac toxicity, liver function test elevation, and interstitial lung abnormality (Table 8).

**TABLE 8:** Adverse events related to treatment.

	*n* = 47
Any grade AE	34 (70.8)
Tachycardia	1 (2.1)
Chest pain	2 (4.2)
Dizziness	3 (6.3)
Vomiting	4 (8.3)
Thrombocytopenia	2 (4.2)
Diarrhea	10 (21.3)
Skin reaction	14 (29.8)
Peripheral neuropathy	10 (20.8)
Cardiac toxicity	1 (2.1)
LFT elevation	1 (2.1)
ILD	1 (2.1)

Abbreviations: AE, adverse event; ILD, interstitial lung disease; LFT, liver function test.

### Next‐generation sequencing (NGS) profile

A total of 14 patients underwent NGS tests of the biopsied lung sample at the time of lazertinib initiation. A total of seven (50%) patients showed T790M mutation, and four patients showed TP53 (28.6%) (Table S1). Patients with TP53 showed a trend toward worse PFS rate when compared to patients who did not have TP53, but no statistical significance was present.

## DISCUSSION

The median PFS of the patients was 15.4 months (95% CI: 8.7–22.1 months), longer than that of a previous recent study, in which median PFS was 13.9 (11.0‐NR) months,^14^ and longer than that of the LASER 201 study of 11.1 (95% CI: 5.5–16.4) months.^9^ Possible reasons for this longer median PFS are a smaller percentage of brain metastases at diagnosis among the enrolled patients (18.7%) and a larger percentage of patients diagnosed with stage III disease (6.2%). The 6‐ and 12‐month PFS in our study were 79.1% and 53.6%, respectively, marginally higher than those reported in the LASER 201 study results, which were 59.3% (46.9–69.8) at 6 months and 48.0% (35.6–59.3) at 12 months. Nonetheless, the 18‐month survival rate in our study was lower than that in the LASER 201 study (27.3% vs. 38%).

An analysis of subgroups stratified by type of *EGFR* mutation at diagnosis showed no significant difference between patients with the L858R mutation and those with the exon 19 deletion. Although the subgroup with the exon 19 deletion initially showed a tendency for higher PFS at the six‐ and 12‐month mark, the groups converged to show no difference in the 18‐month PFS. This convergence is also shown in the Kaplan–Meier analysis. Similarly, a recent study by Kim et al. reported no significant difference in PFS between the two *EGFR* mutation subgroups.^15^ However, it is possible that a future study with a larger sample size and reduced heterogeneity may show different results. Notably, the LASER 301 study revealed a longer median PFS for patients with the exon 19 deletion mutation (20.7 months) compared to those with the L858R mutation (17.8 months), even though the study aimed to assess the efficacy of lazertinib as a first‐line treatment.^7^

In our multivariate analysis for PFS, prior TKI duration and ECOG at lazertinib initiation were independent prognostic factors. In our OS analysis, the ECOG score was the sole independent prognostic factor. In a study on the use of osimertinib after progression on prior EGFR TKI treatments, a shorter duration of prior EGFR‐TKI therapy was statistically associated with poor outcome. Those authors suggested that early resistance to first‐ and second‐generation EGFR‐TKI might accompany other concurrent genetic alterations, in addition to T790M, which could contribute to accelerated resistance to osimertinib.^16^

In the subgroup analysis of NGS results obtained from samples acquired after progression on prior treatment, no significant differences in outcomes were shown when stratified by comutations and TP53 mutations. While there was a trend toward poorer PFS rates in groups with other comutations on NGS, the difference was not significant, possibly due to the small cohort size. Given that concurrent comutations are expected to induce resistance to TKI treatment,^4^, ^17^ a more comprehensive evaluation of NGS results in a future study with a larger sample size is needed.

In terms of safety, lazertinib showed general tolerability. The AEs most commonly reported were skin reactions, diarrhea, and peripheral neuropathy. Notably, the prevalence of peripheral neuropathy in our study was higher in comparison with other targeted treatments. While this is not depicted in the results, most patients who experienced peripheral neuropathy continued the treatment, with some undergoing dose adjustments. To enhance patient adherence and to sustain therapeutic benefits, effective strategies to mitigate peripheral neuropathy are necessary.

The present study had several limitations. First, the short duration of lazertinib usage resulted in a limited number of patients in the analysis. This suggests that increasing the patient sample in future studies may change the risk factor assessment. Second, the study did not evaluate the effects of dose reduction. However, a previous study of lazertinib as a second‐line treatment or beyond did not identify dose modification as an independent prognostic factor.^15^ Third, a larger cohort data will also suggest relative influence of prior first‐generation or second‐generation TKI therapy on the efficacy outcomes with lazertinib. Lastly, in this study, the cohort with available NGS data at the initiation of lazertinib treatment was limited to 14 patients, limiting an accurate comparative of survival analyses among those with and without specific comutations. A future prospective study with expanded cohorts are necessary to more definitively show the prognostic implications of comutations in this context.

In conclusion, in patients with advanced and metastatic NSCLC who progressed after prior EGFR TKI treatment, lazertinib was effective and well tolerated when administered as a second‐line therapy. Thus, we conclude that lazertinib may play an important clinical role in the treatment of patients with advanced NSCLC with *EGFR* mutation. The long‐term outcomes of ongoing trials will provide a more detailed understanding of this promising treatment option.

## AUTHOR CONTRIBUTIONS

All authors had full access to the data in the study and take responsibility for the integrity of the data and the accuracy of the data analysis. Conceptualization: Jeong Uk Lim. Methodology: Jeong Uk Lim. Investigation: Jeong Uk Lim Kyuhwan Kim, Kyu Yean Kim, Hye Seon Kang, Ah Young Shin, Chang Dong Yeo, Sung Kyoung Kim, Chan Kwon Park, Sang Haak Lee and Seung Joon Kim. Formal analysis: Jeong Uk Lim, Chang Dong Yeo and Seung Joon Kim. Resources: Jeong Uk Lim. Writing—original draft: Jeong Uk Lim, Chang Dong Yeo and Seung Joon Kim. Writing—review and editing: All authors. Visualization: Jeong Uk Lim. Supervision: Chang Dong Yeo and Seung Joon Kim. Funding acquisition: Jeong Uk Lim. Jeong Uk Lim is guarantor of the content of the manuscript including the data and analysis. Jeong Uk Lim, Chang Dong Yeo and Seung Joon Kim conceived and designed the study. Jeong Uk Lim, and Chang Dong Yeo performed the statistical analysis.

## CONFLICT OF INTEREST STATEMENT

The authors confirm there are no conflicts of interest.

## Supporting information


**TABLE S1.** Next‐generation sequencing test results at the time of lazertinib initiation.
