# Supplementary material for: Real‐world study of lazertinib as second‐line or greater treatment in advanced non‐small cell lung cancer
Source: Thorac Cancer. 2024 May 27;15(19):1513–21. doi: 10.1111/1759-7714.15337 (PMC11219289; doi:10.1111/1759-7714.15337)
Supplement: Supplementary file 1 — TABLE S1. Next‐generation sequencing test results at the time of lazertinib initiation. [file TCA-15-1513-s001.docx]

**Supplementary Table 1. Next-generation sequencing test results at the time of lazertinib initiation**

| **Characteristics** | **Total (n=14)** |
| --- | --- |
| T790M mutation | 7 (50%) |
| Co-mutation from NGS | 8 (57.1%) |
| TP53 | 4 (28.6%) |
| PIK3CA | 2 (14.3%) |
| Exon 20 point mutation | 1 (7.2%) |
| Other co-mutations detected (One patient per each mutation) | TET2, RB1, ERBB2 amplification, CDK4, FGF23, MYC, MDM2, CCND2 amplification |

**Abbreviation: NGS, Next-Generation Sequencing; TP53, Tumor Protein p53; PIK3CA, Phosphatidylinositol-4,5-Bisphosphate 3-Kinase Catalytic Subunit Alpha; ERBB2, Erb-B2 Receptor Tyrosine Kinase 2; CDK4, Cyclin-Dependent Kinase 4; FGF23, Fibroblast Growth Factor 23; MYC, MYC Proto-Oncogene; CCND2 - Cyclin D2**
